# Entropic Uncertainty in Spin XY Model with Long-Range Interactions

**DOI:** 10.3390/e22080837

**Published:** 2020-07-30

**Authors:** Nour Zidan

**Affiliations:** 1 Mathematics Department, College of Science, Jouf University, P.O. Box 2014 Sakaka, Saudi Arabia; nazidane@ju.edu.sa; 2 Mathematics Department, Faculty of Science, Sohag University, P.O. Box 82525 Sohag, Egypt

**Keywords:** uncertainty relations, Sutherland–Calogero–Moser model, entanglement, mixedness

## Abstract

The behavior of the uncertainty relations and their tightness for a system, consisting of two qubits interacting thermally with a magnetic field in the presence of Dzyaloshinskii–Moriya interaction, is discussed, where different types of interaction strengths are considered. It is shown that both coupling and the magnetic field parameters decay the degree of entanglement, and increasing the uncertainty relations and the degree of mixedness. The phenomena of the sudden changes in the investigated quantities are depicted at large values of the field and coupling parameters. Concerning the type of the coupling parameters, distance and the trigonometric coupling have a clear effect on the behavior of the studied physical quantities.

## 1. Introduction

The uncertainty principle is one of the most remarkable characters of quantum mechanics as well as a fundamental departure from the principle of classical physics [1,2,3]. Any pair of incompatible observables complies with a certain form of uncertainty relationship, the constraint of which sets ultimate bounds on the measurement precision for these quantities and provides a theoretical basis for new technologies, such as quantum cryptography in quantum information [4,5,6,7]. The new entropic uncertainty principle has been recently confirmed experimentally [8,9] and ignites the interest of people to investigate its potential applications from various aspects [10,11]. More recently, a new type of Heisenberg relation, i.e., the quantum memory assisted entropic uncertainty relation, has been derived [12] according to the conjecture of Renes and Boileau [13]. Owing to its versatile applications, entropic uncertainty relation can be potentially applied to quantum key distribution [14,15], probing quantum correlations [16,17,18,19,20], quantum randomness [21], cryptographic security [22,23], entanglement witness [24,25,26,27,28,29], and quantum metrology [30,31,32]. It is worth mentioning that the close relation between mixedness and uncertainty has been discussed widely as a concerned topic [33,34,35,36,37]. The dynamics of entropic uncertainty relation in a Heisenberg spin chain, under an inhomogeneous magnetic field, has been explored [38,39,40]. Thermal quantum correlations and the entropic uncertainty relation in the presence of quantum memory under two kinds of two-qubit spin squeezing models have been investigated [41]. On other hand, a new type of long-range reaction has been used in the references [42,43] to obtain long-distance entanglement in the spin system. In these works, the spin pair reaction is given by a factor that is inversely proportional to the strength of the distance between locations such as $J\left(r\right)∼r^{-\alpha }$. In these studies it is shown that long-distance entanglement can be obtained by using this type of reaction with different values of the α reaction parameter in Heisenberg spin systems. Indeed, the inverse-square, trigonometric and hyperbolic interacting particle systems [44,45,46] and its spin generalizations [47,48] are an important model of many-body systems. These interaction types are called Sutherland–Calogero–Moser (SCM) model or SCM type interactions. Meanwhile, many studies show that the long-range interaction plays an important role in quantum information [49,50,51]. Moreover, the entanglement under the effect of long-range interaction has been investigated [52,53,54,55,56]. Also, the possibility of using the long-range interaction of spin models in quantum communication has been illustrated [57,58,59,60]. In this work, we invoke a new perspective on the entropic uncertainty relation for the Heisenberg XY model with long-range interaction. We focus on the fact that parameters such as spin interaction coupling, magnetic field, and Dzyaloshinskii–Moriya interaction, may be used as control factors to show the effect on uncertainty relation and its tightness, concurrence, and mixedness. The outline of this paper is as follows. In Section 2, the suggested system and its time evolution are introduced as well as the definitions of some physical quantities, which are in our pivot of interest, such as uncertainty, concurrence, and mixedness. Section 3 is devoted to discussing the behaviors of these quantities numerically at different initial values of the Dzyaloshinskii–Moriya interaction, the strength of the magnetic field and the temperature. Conclusions are given in Section 4.

## 2. The Suggested Model

In this work, we consider the Hamiltonian of the Heisenberg XY chain in the presence of a uniform magnetic field and Dzyaloshinskii–Moriya interaction in the form [52]:

(1) $$H=b\left\{\sigma_{1}^{z}+\sigma_{2}^{z}\right\}-J\left(r\right)\left\{\sigma_{1}^{x}\sigma_{2}^{x}+\sigma_{1}^{y}\sigma_{2}^{y}\right\}+D\left\{\sigma_{1}^{x}\sigma_{2}^{y}-\sigma_{1}^{y}\sigma_{2}^{x}\right\},$$

where *b* is the uniform magnetic field, *D* is the Dzyaloshinskii–Moriya (DM) strength and $\sigma_{j}\left(\sigma_{j}^{x},\sigma_{j}^{y},\sigma_{j}^{z}\right)$ is the vector of Pauli matrices. Here the J(r) denotes the spin interaction coupling, which will be defined in terms of Sutherland-Calogero–Moser (SCM). On the standard basis $\left\{|00\rangle ,|01\rangle ,|10\rangle ,|11\rangle \right\}$ the Hamiltonian (1) can be calculated in the matrix form as:

(2) $$H=\left(\begin{array}{cccc} 2b & 0 & 0 & 0\\ 0 & 0 & -2(J(r)-iD) & 0\\ 0 & -2(J(r)+iD) & 0 & 0\\ 0 & 0 & 0 & -2b \end{array}\right).$$

By diagonalized the above Hamiltonian, the corresponding eigenvalues and eigenvectors for all types of J(r) are given by

(3) $$E_{1,2}=\pm 2b,\;\;E_{3,4}=\pm 2\sqrt{J{\left(r\right)}^{2}+D^{2}},$$

and

(4) $$\begin{array}{ccc} |\psi_{1}\rangle & = & |11\rangle ,\;\;\;\;|\psi_{2}\rangle =|00\rangle , \end{array}$$

(5) $$\begin{array}{ccc} |\psi_{3}\rangle & = & \frac{1}{\sqrt{2}}\left(-exp\left[-i\theta \right]|10\rangle +|01\rangle \right), \end{array}$$

(6) $$\begin{array}{ccc} |\psi_{4}\rangle & = & \frac{1}{\sqrt{2}}\left(exp\left[-i\theta \right]|10\rangle +|01\rangle \right), \end{array}$$

where θ=arc$\tan\left[\frac{D}{J(r)}\right]$. For a spin system in equilibrium at temperature *T*, the thermal state density matrix is $\rho \left(T\right)=\frac{1}{Z}\exp\left[-\frac{H}{kT}\right]=\frac{1}{Z}\Sigma_{i=1}^{4}exp\left[-E_{i}/kT\right]|\psi_{i}\rangle \langle \psi_{i}|$ where $Z=Tr\left[\exp\left[-\frac{H}{kT}\right]\right]$ is the partition function and *k* is Boltzmann constant (we will use a unit system where the Boltzmann constant k=1). In the standard basis above-mentioned, after some calculation we can obtain the density matrix in the form:

(7) $$\rho \left(T\right)=\frac{1}{Z}\left(\begin{array}{cccc} \rho_{11} & 0 & 0 & 0\\ 0 & \rho_{22} & \rho_{23} & 0\\ 0 & \rho_{32} & \rho_{33} & 0\\ 0 & 0 & 0 & \rho_{44} \end{array}\right),$$

where

$$\begin{array}{ccc} \rho_{11} & = & \exp\left[-2b/T\right],\;\;\;\rho_{44}=\exp\left[2b/T\right],\\ \rho_{22} & = & \rho_{33}=\cosh\left[2\sqrt{J{\left(r\right)}^{2}+D^{2}}/T\right],\\ \rho_{23} & = & \rho_{32}^{*}=iexp\left[-i\theta \right]\sinh\left[2\sqrt{J{\left(r\right)}^{2}+D^{2}}/T\right],\\ Z & = & 2\left(\cosh\left[2b/T\right]+\cosh\left[2\sqrt{J{\left(r\right)}^{2}+D^{2}}/T\right]\right). \end{array}$$

## 3. Entropic Uncertainty and Correlation Measures

In this section, we investigate the dynamic of four different quantities of a two-qubit system; the entropic uncertainty, tightness of the uncertainty, entanglement, and mixedness.

- *The entropic uncertainty*
  This quantity may be described by the following scenario:
  We consider two users Alice and Bob who play a game, where the second player, Bob, prepares a qubit in a quantum state of his choice and sends it to the first player, Alice, who performs one of two measurements and announces her choice to Bob. Based on the received measurement, Bob can minimize his uncertainty. The uncertainty relation is expressed in terms of the standard deviation $\Delta M\;\Delta N\geq \frac{1}{2}\left|\langle \left[M\;,\;N\right]\rangle \right|$ for two observables *M* and *N* [2,61]. Instead of standard deviation, Deutsch [62] quantified uncertainty in terms of Shannon entropy and derived the entropic uncertainty relation for any pair of observables [63]. Later Maassen and Ufnk [64] improved Deutsch’s job and gave the following tighter entropic uncertainty relations:
  (8) $$S\left(M\right)+S\left(N\right)\geq \log_{2}\frac{1}{c},$$
  where S(M) denotes the Shannon entropy of the probability distribution of the outcomes when *M* is measured, and likewise for S(N). 1/c quantifies the complementarity of *M* and *N*, where $c=max_{m,n}{\left|\langle \psi_{m}|\phi_{n}\rangle \right|}^{2}$ for nondegenerate observables, with $|\psi_{m}\rangle$ and $|\phi_{n}\rangle$ being the eigenvectors of *M* and *N*. Very recently, a quantum memory assisted entropic uncertainty relation has been proposed [12,13] and experimentally demonstrated [8,9], which reads
  (9) $$S\left(M|B\right)+S\left(N|B\right)\geq S\left(A|B\right)+\log_{2}\frac{1}{c},$$
  where $S\left(A|B\right)=S\left(\rho_{AB}\right)-S\left(\rho_{B}\right)$ is the conditional von Neumann entropy. After, the qubit *A* is measured by *M*, where the post-measurement state is
  (10) $$\rho_{AB}=\sum_{i}\left({|\psi_{i}\rangle }_{A}\langle \psi_{i}|⊗I_{B}\right)\rho_{AB}\left({|\psi_{i}\rangle }_{A}\langle \psi_{i}|⊗I_{B}\right).$$
- *Tightness*
  For convenience, we use *R* and *L* to denote the right and the left side of the uncertainty relation in Equation (9), and define U=L−R as the tightness of the uncertainty relation, which measures the discrepancy of the right and the left sides of the inequality Equation (9).
- *Entanglement*
  Entanglement plays a central role in quantum computation and quantum information. There are several measures to quantify the amount of entanglement contained in a quantum system. In this treatment, we consider the concurrence [65] as the accepted and common measure of the two-qubit system. However, for any state $\rho_{ab}$, the concurrence is defined
  (11) $$C=max\left[0,2max\left[\lambda_{i}\right]-\sum_{i=1}^{4}\lambda_{i}\right],$$
  where the quantities $\lambda_{i}\left(i=1,2,3,4\right)$ are the square roots of the four eigenvalues of the matrix $\Gamma =\rho \Theta \rho^{*}\Theta$, ρ is the density matrix and $\Theta =\sigma_{y}⊗\sigma_{y}$.
- *Mixedness*
  For a density matrix ρ, the state is a pure state if $Tr\left(\rho^{2}\right)=1$, and if $Tr\left(\rho^{2}\right)<1$ for a mixed one. Thus the mixedness $\left(X\right)$ can be defined as [66]:
  (12) $$X=\frac{d}{d-1}\left[1-Tr\left(\rho^{2}\right)\right],$$
  where *d* is the dimension of state ρ.

## 4. Numerical Discussion

In this section, we will discuss the dynamics of the uncertainty, tightness of the uncertainty, entanglement, and mixedness for the XY model (1), where we consider different types of coupling interaction, which are defined in the Sutherland–Calogero–Moser model [44,45,46]. Therefore, in this regard, we shall consider the inverse square interaction coupling $J_{0}/r^{2}$ and trigonometric interaction coupling $J_{0}csc^{2}\left(r\right)$.

### 4.1. The Inverse-Square Type

Firstly, we consider the inverse-square interaction version of long-range interaction in the Sutherland–Calogero–Moser model, which is defined with exchange interaction $J\left(r\right)=J_{0}/r^{2}$ (set $J_{0}=1)$ [44,45,46].

**Figure 1:**
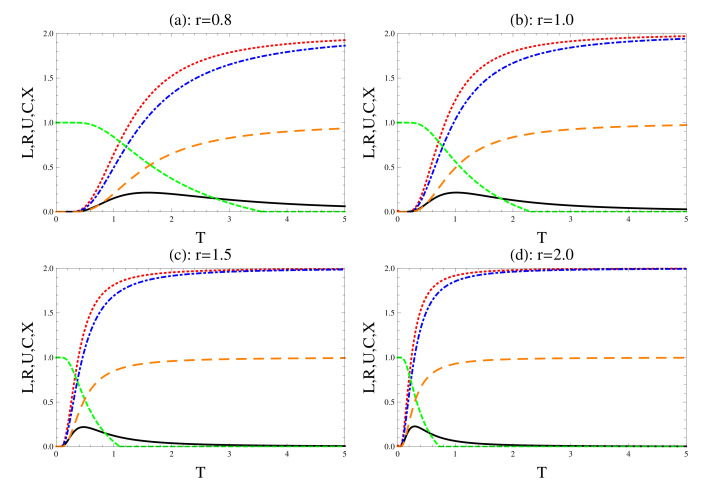
The effect of the spin coupling *r*, where we set D=0.2 and b=0.1. The dot (red), the dash-dot (blue) and the solid (black) for the left and right uncertainty relations and their tightness, respectively. The short (green)/long dashes (brown) for the concurrence and mixedness, respectively.

In Figure 1, we investigate the effect of the coupling distance on the behavior of all the physical quantities, uncertainty and its tightness, concurrence and the degree of the mixedness as a function of the temperature *T*. Different values of the parameter *r* are considered, while the other values of the parameters are fixed, namely, we set the Dzyaloshinskii–Moriya parameter, D=0.2, and the strength of the magnetic field b=0.1. It is clear that both sides of the uncertainty relation (left and right hand sides; LHS,RHS, respectively) increase as the temperature increases. However, as the *T* increases further, the uncertainty tends to a fixed maximum. Moreover, the behavior of the uncertainty reveals that LHS>RHS for all *T*. The behavior of the tightness shows that the variation between the LHS and the RHS of the uncertainty inequality decreases as the temperature increases. The decoherence is displayed clearly on the behavior of the concurrence and the mixedness degree, where the concurrence, C decreases fast as the temperature increases and completely vanishes at T>3.6. This means that the classical correlation increases on the expanse of the quantum correlation and the initial entangled system becomes separable. These results are confirmed from the behavior of the mixedness, where *X* increases as the *T* increases. The effect of the parameter *r* on these quantities appears clearly by comparing Figure 1a–d, whereas *r* increases, namely the coupling decreases, the uncertainty relations, *R* and *L* increase while their tightness decreases. The degree of entanglement and mixedness behaves differently as *r* increases, the concurrence decreases to vanish completely at a different temperature depending on the initial *r*. Meanwhile, the mixedness degree increases gradually to reach its maximum bounds quickly as *r* increases.

**Figure 2:**
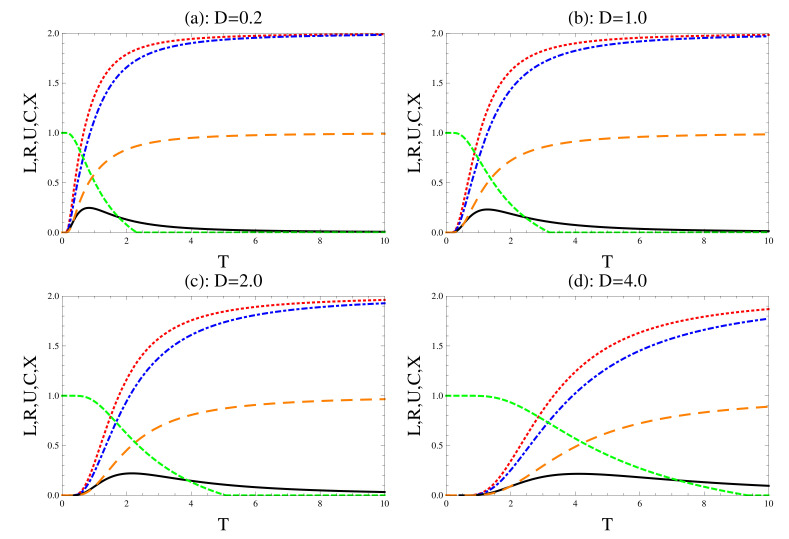
The effect of the Dzyaloshinskii–Moriya, where we set r=1.0 and b=0.5. The dot (red), the dash-dot (blue) and the solid (black) for the left and right uncertainty relations and their tightness, respectively. The short (green)/long dashes (brown) for the concurrence and mixedness, respectively.

The effect of DM interaction on the uncertainty relations, concurrence and the degree of mixedness is displayed in Figure 2, where different values of the interaction’s strength are considered. In general, the behavior is similar to that displayed in Figure 1. However, larger values of DM’s strength increase the quantum correlation between the two qubits, where the concurrence increases as the *T* increases at large values of *D*. Larger values of DM interaction increases the life of the survival amount of quantum correlations as *T* increases. The fast/sudden decay of the concurrence turns into gradually/nonvanishing at large values of *D*. The upper bounds of uncertainty relations and their tightness decrease/increase as DM strength increases. Moreover, the degree of violating these relations increases as DM increases, namely the quantum correlation increases. Comparing the behavior of the degree of mixedness in Figure 2a–d, one may conclude that *X* decreases as DM strength increases, namely the degree of decoherence decreases.

**Figure 3:**
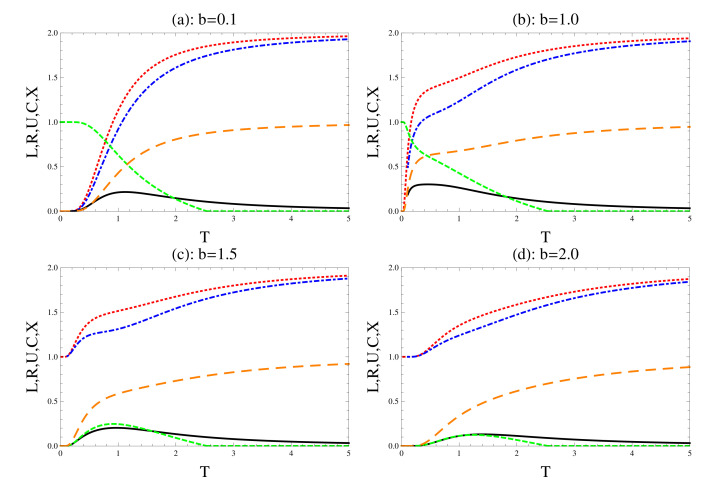
The effect of the spin coupling *b*, where we set D=0.5 and r=1.0. The dot (red), the dash-dot (blue) and the solid (black) for the left and right uncertainty relations and their tightness, respectively. The short (green)/long dashes (brown) for the concurrence and mixedness, respectively.

In Figure 3, we touch on the effect of the magnetic field strength on the previous physical quantities that are the focus of our study, where the other parameters are assumed to be fixed. As it is displayed, from Figure 3a, where the small value of the magnetic field is settings (b=0.1), the uncertainty relations are obeyed at a small temperature, (T<0.2) and consequently, the system contains only quantum correlation. As the temperature increases further, the uncertainty relations are violated and increase gradually, namely, the system loses its quantum coherence. At larger values of the magnetic strength, the uncertainty relations and their tightness increase suddenly to reach their maximum bounds, while the concurrence decreases gradually as the temperature increases.

### 4.2. Trigonometric Coupling

**Figure 4:**
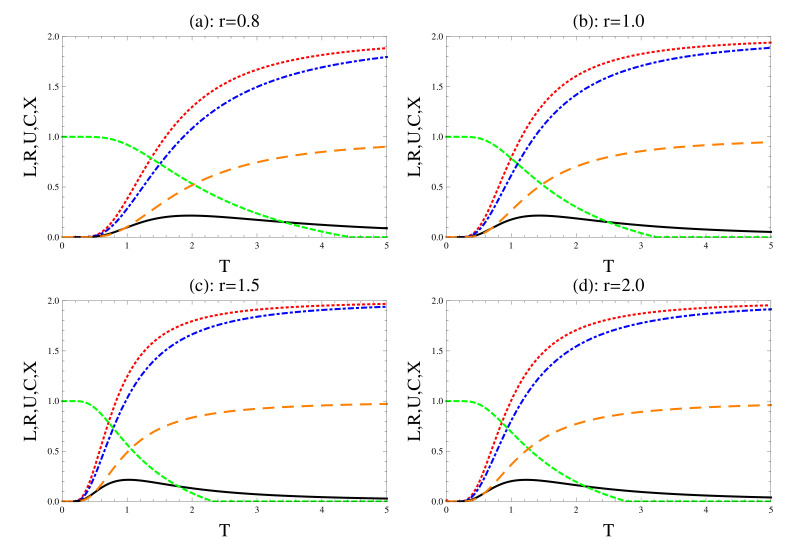
The same as Figure 1, but the coupling constant is defined by trigonometric type, $J_{0}csc^{2}\left(r\right)$.

Finally, we consider the trigonometric version of long-range interaction in the Sutherland–Calogero–Moser model, which is defined with exchange interaction $J\left(r\right)=J_{0}csc^{2}\left(r\right)$ (set $J_{0}=1)$ [44,45,46]. As it is displayed from Figure 4, the general behavior of the uncertainty relation and their tightness are similar to that displayed in Figure 1. As it is displayed from Figure 4a, the entanglement is maximum, namely the concurrence C=1 at a small temperature. Meanwhile, the other physical quantities have zero values. It is clear that by comparing Figure 1 and Figure 4, one can see that the decay rate of all the physical quantities is larger than that displayed in Figure 1, where the entanglement vanishes at smaller temperatures, the uncertainty relations increase faster and reach their maximum values at a small temperature. The uncertainty relations are obeyed at a small temperature, where these results are depicted by comparing the degree of tightness that is displayed in Figure 1 and Figure 4.

**Figure 5:**
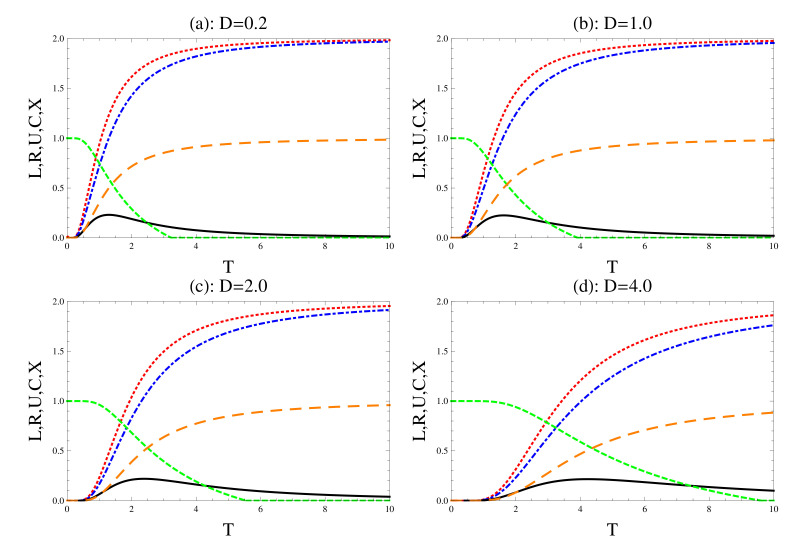
The same as Figure 2, but the coupling constant is defined by trigonometric type, $J_{0}csc^{2}\left(r\right)$.

In Figure 5, we investigate the effect of the DM interaction when the coupling is given by the hyperbolic function, where we fixed the coupling function and the strength of the magnetic field parameter. It is clear that the behavior is similar to that displayed in Figure 2, namely, the large values of DM strength increase the degree of entanglement and increase the robustness of quantum correlation at large temperatures. Moreover, by comparing Figure 2 and Figure 4, one may conclude that the type of coupling plays an important role in the increasing/decreasing rate of all the physical quantities. Moreover, for the trigonometric coupling, the DM interaction increases the violation degree of the uncertainty relations.

**Figure 6:**
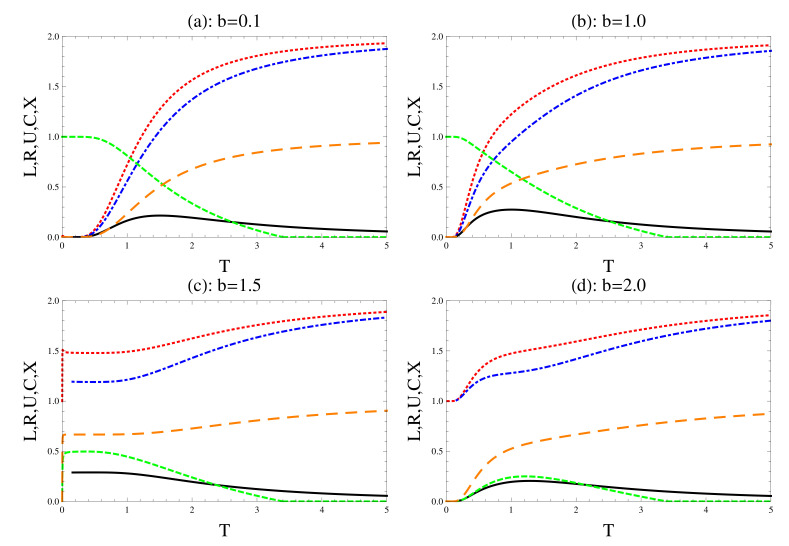
The same as Figure 3, but the coupling constant is defined by trigonometric type, $J_{0}csc^{2}\left(r\right)$.

Finally, we discuss the effect of the magnetic field strength *b* in the presence of the trigonometric coupling, where we set the same values as considered in Figure 3. As it is displayed from Figure 6, the behavior of all the physical quantities is similar to that displayed in Figure 3. However, by comparing all of Figure 3 and the corresponding graphs of Figure 6, the entanglement decays faster than those displayed in Figure 6. Similarly, when the magnetic field increases, the rate of increase of uncertain relationships increases, and the rate of this increase shown in Figure 3 is smaller than shown in Figure 6. The degree of mixedness increases as the strength *b* increases, where the increasing rate displayed in Figure 6 is slightly larger than that shown in Figure 1. Also, as it is displayed from Figure 3, the degrees of tightness are larger than their corresponding values in Figure 6.

## 5. Conclusions

In this contribution, we investigate different quantum physical quantities, which may be considered as indicators of coherence and decoherence of an entangled quantum system. In this context, we consider partial anisotropy qubits governed by an XY-Heisenberg chain model in the presence of a Dzyaloshinskii–Moriya interaction and magnetic field. The effect of the initial parameters of the suggested Hamiltonian on the uncertainly relations and their tightness as well as on the degree of entanglement and mixedness is discussed.

It is shown that the coupling spin interaction has a clear effect on the decoherence properties of the entangled state, where two different types of coupling are considered; distance and trigonometric couplings. It is shown that the type of coupling has a clear effect on the classical and quantum correlation of the system. At fixed values of Dzyaloshinskii–Moriya and the magnetic field strength, the entanglement decays fast as the temperature increases, meanwhile, the uncertainty relations and the mixedness degree increase. However, as one increases the coupling parameter, the phenomena of the sudden death and the sudden increase of the concurrence and the uncertainty relation, respectively, appear. Moreover, the degree of tightness decreases faster and completely vanishes at larger values of the coupling at a smaller temperature. This means that the uncertainty relations are obeyed and the system loses its quantum correlation and turns into a separable system.

The effect of the Dzyaloshinskii–Moriya on the investigated quantities predicts a different behavior, where it is shown that the larger values of DM interaction improve the amount of quantum correlation. Meanwhile, the degree of obeying the uncertainty relation decrease as DM increases, i.e., the degree of the obstacle of the systems against the decoherence increases. Moreover, at larger values of DM, the sudden behaviors of all the studied quantities disappear.

The parameter of the magnetic field has a similar effect of the coupling parameter, but the degree of decoherence of the entanglement is smaller than that displayed at larger values of the coupling parameter. However, the increasing rate of the uncertainty relations is smaller than that predicted for the effect of the coupling parameter. The degree of tightness is larger than that displayed for the coupling parameter.

Concerning the type of the coupling parameters; distance and the trigonometric coupling have a clear effect on the behavior of the studied physical quantities. The behavior of all the physical quantities is the same for both types of coupling. However, for the rate of decay/increasing these phenomena depicted for the trigonometric coupling is faster/smaller than that displayed in the presence of the distance coupling.
